# Outcome pre-specification requires sufficient detail to guard against outcome switching in clinical trials: a case study

**DOI:** 10.1186/s13063-018-2654-z

**Published:** 2018-05-02

**Authors:** Brennan C. Kahan, Vipul Jairath

**Affiliations:** 1 Pragmatic Clinical Trials Unit, Queen Mary University of London, 58 Turner St, London, E1 2AB UK; 2 Department of Medicine, Division of Gastroenterology, University Hospital, London, ON Canada; 3 Department of Epidemiology and Biostatistics, Western University, London, ON Canada

**Keywords:** Selective outcome reporting, Outcome reporting bias, Clinical trial

## Abstract

**Background:**

Pre-specification of outcomes is an important tool to guard against outcome switching in clinical trials. However, if the outcome is not sufficiently clearly defined, then different definitions could be applied and analysed, with only the most favourable result reported.

**Methods:**

In order to assess the impact that differing outcome definitions could have on treatment effect estimates, we re-analysed data from TRIGGER, a cluster randomised trial comparing two red blood cell transfusion strategies for patients with acute upper gastrointestinal bleeding. We varied several aspects of the definition of further bleeding: (1) the criteria for what constitutes a further bleeding episode; (2) how further bleeding is assessed; and (3) the time-point at which further bleeding is measured.

**Results:**

There were marked discrepancies in the estimated odds ratios (OR) (range 0.23–0.94) and corresponding *P* values (range < 0.001–0.89) between different outcome definitions. At the extremes, differing outcome definitions led to markedly different conclusions; one definition led to very little evidence of a treatment effect (OR = 0.94, 95% confidence interval [CI] = 0.37–2.40, *P* = 0.89), while another led to very strong evidence of a treatment effect (OR = 0.23, 95% CI = 0.11–0.50, *P* < 0.001).

**Conclusions:**

Outcomes should be pre-specified in sufficient detail to avoid differing definitions being analysed and only the most favourable result being reported.

**Trial registration:**

Clinical Trials.gov, NCT02105532. Registered on 7 April 2014.

## Background

Randomised controlled trials (RCTs) are the gold standard for assessing new healthcare interventions. An important aspect in the good conduct of RCTs is pre-specification of the primary and secondary outcomes, as this helps to guard against outcome switching. An example of outcome switching includes discarding non-significant outcomes in favour of outcomes with better results [1–12]. Previous reviews have found that primary outcome switching affects up to 67% of trials [1, 2, 5, 8, 9, 13] and, as such, it is widely recommended that all outcomes are listed in the protocol and on a clinical trial registry website before the start of the study.

However, simply listing the trial outcome in the protocol is not sufficient to guard against outcome switching. If the outcome is not sufficiently clear or well-defined in the protocol, investigators could compare results from several modifications of the definition and then present the most favourable. For example, altering the outcome definition or instrument used to measure it, the time-point the outcome is measured at or the method of assessing the outcome, could all lead to different results. The SPIRIT (Standard Protocol Items: Recommendations for Interventional Trials) guidelines [3] and ClinicalTrials.gov registry guidelines [14] require outcome definitions to include elements such as the time-point, measurement, analysis metric, method of aggregation and method of assessment.

However, studies have shown that adherence is not always high and that sufficient detail is often lacking ([14], www.COMPare-trials.org). For example, the COMPARE project found trials which failed to pre-specify the cut-off point for a dichotomous outcome based on a continuous measurement, which domain of a questionnaire would be used or the time-point for assessment of the outcome (www.COMPare-trials.org). Given that varying different elements of the outcome definition could lead to a very large number of potential outcomes [15], providing insufficient detail regarding outcome definitions in trial protocols could allow investigators to apply and analyse different definitions and report only the most favourable result. We aimed to investigate to what extent modifying an outcome definition could influence treatment effect estimates through re-analysis of a previously published trial.

## Methods

### TRIGGER trial

#### Overview

We discuss issues surrounding a clinical outcome definition in the context of the Transfusion in Gastrointestinal Bleeding (TRIGGER) trial [16–18]. TRIGGER was a cluster-randomised feasibility trial which compared two red blood cell (RBC) transfusion strategies for patients with acute upper gastrointestinal bleeding. Patients in hospitals randomised to the liberal transfusion strategy received a RBC transfusion when their haemoglobin dropped below 10 g/dL and patients in hospitals randomised to the restrictive transfusion strategy received a RBC transfusion when their haemoglobin dropped below 8 g/dL.

The primary clinical outcome was further bleeding, which is typically defined as either continued bleeding at the end of the patient’s initial examination (usually using a fibre optic telescope, called an endoscopy) or a bleed that has restarted after initially stopping. The definition was informed by international consensus criteria [19]. However, there are several aspects of further bleeding that need to be clearly specified in order to have a sufficiently clear definition, upon which there is no clear consensus, and various permutations have been used within clinical trials. These are:

- the criteria for what constitutes a further bleeding episode;
- how further bleeding will be measured or assessed;
- who will measure or assess further bleeding;
- the time-point at which the outcome will be measured.

We discuss each of these issues in turn.

#### Criteria for determining whether a further bleeding episode occurred

This involves deciding which criteria will be used to determine whether a further bleeding event has occurred. Several different criteria could be used; for example, the criteria could include both persistent bleeding (bleeding that continues at the end of a patient’s initial examination) and recurrent bleeding (bleeding that has stopped at the end of the initial examination, but which starts again afterwards) or just recurrent bleeding. Additionally, different sets of criteria could be used to determine whether either recurrent or persistent bleeding had occurred. For example, the criteria could be as simple as visualising any blood in the upper gastrointestinal tract or it could be stricter and require that the bleeding be from a particular source, such as a lesion with high-risk stigmata of bleeding (e.g. a peptic ulcer with a visible vessel or overlying clot suggestive of recent bleeding).

#### How further bleeding will be measured or assessed

This involves deciding what information will be used to determine whether the criteria for further bleeding have been met. Assessment of further bleeding could be based upon several different sources of information. It could be based upon some combination of a patient’s physical signs and symptoms, for example, haemodynamic instability such as low blood pressure and an increased heart rate, a rapid drop in the patient’s haemoglobin level, whether the patient has vomited blood or the passage of altered blood per rectum. Conversely, it could be based upon a direct visual inspection of the patient’s upper gastrointestinal tract to confirm the presence of ongoing bleeding from a particular source (e.g. a high-risk stigmata) during an endoscopy or other medical procedure.

#### Who will measure or assess further bleeding

This involves deciding who will assess the information collected to determine whether the criteria for an outcome event has been met. Depending on which information will be used to measure or assess the outcome, a number of different assessors could be used. For example, if the assessment is based solely upon simple patient symptoms such as vomiting blood, this could be self-reported by the patient, assessed by the attending clinician or determined by an independent adjudication committee. If the assessment is based upon a direct visual inspection of the patient’s upper gastrointestinal tract (e.g. via endoscopy), this could be assessed by the clinician performing the endoscopy or by an independent adjudication committee if the endoscopy is recorded.

#### The time-point at which the outcome will be measured

This involves deciding the time-frame that the outcome will be measured within. For example, further bleeding could be measured while the patient remains in hospital or after a set amount of time since randomisation, such as within 28 days.

### Re-analysis of TRIGGER to assess impact of different outcome definitions on results

We explored what impact modifying the definition of further bleeding in TRIGGER could have on the trial results. Based on the data collected during the trial, we were able to vary several elements of the outcome definition to explore these post-hoc analyses:

- *Definition of further bleeding*: persistent and recurrent bleeding vs recurrent bleeding only;
- *Method of assessment*: direct visual inspection of the upper gastrointestinal tract during endoscopy/surgery/radiology vs direct visual inspection or clinician judgement based on patient symptoms;
- *Time-point*: in-hospital vs up to day 28.

This led to eight different outcome definitions (one for each combination above). The main outcome specified in the TRIGGER trial itself was persistent and recurrent bleeding up to 28 days, assessed via direct visual inspection of the upper gastrointestinal tract during endoscopy/surgery/radiology.

We analysed each outcome definition using a logistic regression model with generalised estimating equations [20], with an exchangeable correlation structure within clusters and robust standard errors. The model adjusted for the following covariates: presence of shock; age; the number of co-morbidities; and the presence of coagulopathy [21, 22].

## Results

**Fig. 1:**
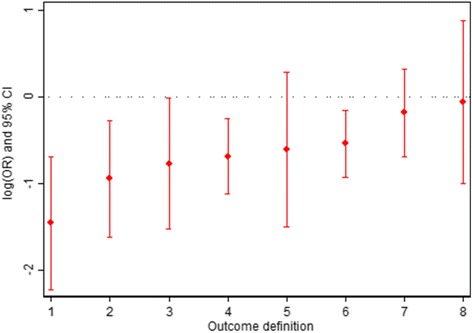
Results for different outcome definitions of further bleeding in TRIGGER. *Outcome definitions: (1) recurrent bleeding only, in hospital, assessed via direct visual inspection; (2) recurrent bleeding only, up to day 28, assessed via direct visual inspection; (3) recurrent bleeding only, in hospital, assessed via clinical judgement or direct visual inspection; (4) recurrent and persistent bleeding, up to day 28, assessed via direct visual inspection; (5) recurrent and persistent bleeding, in hospital, assessed via direct visual inspection; (6) recurrent bleeding only, up to day 28, assessed via clinical judgement or direct visual inspection; (7) recurrent and persistent bleeding, up to day 28, assessed via clinical judgement or direct visual inspection; (8) recurrent and persistent bleeding, in hospital, assessed via clinical judgement or direct visual inspection

Results are shown in Fig. 1 and Table 1. There were marked discrepancies in the estimated odds ratios (OR) (range 0.23–0.94) and corresponding *P* values (range < 0.001–0.89) between different outcome definitions. Results were statistically significant for 5/8 outcome definitions. Estimated ORs were more extreme for outcomes based on recurrent bleeding only vs recurrent and persistent bleeding and for outcomes assessed via direct visual inspection only vs clinical judgement or direct visual inspection.

**Table 1:** Results for different outcome definitions of further bleeding in TRIGGER

Time-point	Method of assessment	Definition	Liberal policy (*n* (%)) (*n* = 403)	Restrictive policy (*n* (%)) (*n* = 533)	Odds ratio^c^(95% CI)	*P* value
In hospital^a^	Clinical judgement or visual inspection	Recurrent and persistent bleeding	31 (5.8)	18 (4.5)	0.94 (0.37–2.40)	0.89
In hospital^a^	Clinical judgement or visual inspection	Recurrent bleeding only	21 (4.0)	8 (2.0)	0.46 (0.22–0.98)	0.04
In hospital^a^	Visual inspection only	Recurrent and persistent bleeding	24 (4.5)	9 (2.2)	0.54 (0.22–1.33)	0.18
In hospital^a^	Visual inspection only	Recurrent bleeding only	14 (2.6)	3 (0.7)	0.23 (0.11–0.50)	< 0.001
Day 28^b^	Clinical judgement or visual inspection	Recurrent and persistent bleeding	42 (8.2)	27 (6.9)	0.83 (0.50–1.37)	0.47
Day 28^b^	Clinical judgement or visual inspection	Recurrent bleeding only	32 (6.3)	17 (4.3)	0.58 (0.39–0.86)	0.007
Day 28^b^	Visual inspection only	Recurrent and persistent bleeding	31 (6.1)	13 (3.3)	0.50 (0.32–0.78)	0.002
Day 28^b^	Visual inspection only	Recurrent bleeding only	21 (4.1)	7 (1.8)	0.39 (0.20–0.76)	0.006

^a^In hospital: 1 patient was excluded from the analysis because of missing data on further bleeding (this left 532 patients in the liberal policy group and 403 in the restrictive policy group)

^b^Day 28: 31 patients were excluded from the analysis because of missing data on further bleeding (this left 512 patients in the liberal policy group and 393 in the restrictive policy group)

^c^Analysis was conducted using generalised estimating equations, with an exchangeable correlation structure within clusters and robust standard errors. The model adjusted for the following covariates: presence of shock; age; the number of co-morbidities; and the presence of coagulation

At the extremes, different outcome definitions led to markedly different conclusions; a definition which included both recurrent and persistent bleeding, measured in hospital, and assessed via clinical judgement or direct visual inspection would have led to very little evidence of a difference between treatment groups (OR = 0.94, 95% confidence interval [CI] = 0.37–2.40, *P* = 0.89), whereas a definition based on recurrent bleeding only, measured in hospital, based on direct visual inspection only would have led to very strong evidence of difference between treatment groups (OR = 0.23, 95% CI = 0.11–0.50, *P* < 0.001).

## Discussion

Pre-specification of outcomes in RCTs is an important tool to guard against outcome switching. Guidelines such as the SPIRIT statement and the ClinicalTrials.gov registry require outcome definitions to include important elements such as the time-point, measurement, metric and method of aggregation. However, research has shown that outcomes are not always defined in sufficient detail, which may enable investigators to analyse several different modifications of the outcome definition and present only the most favourable result.

In our post-hoc re-analysis of the TRIGGER trial, we explored the potential impact that modifications to an outcome definition could have on estimated treatment effects. We found that it was possible to obtain both significant and non-significant results by simply altering the definition of what constituted a bleeding event, the time-point at which further bleeding was measured and the method of assessment. At the extremes, it was possible to obtain either strong evidence of a treatment effect (OR = 0.23, *P* < 0.001) or no evidence of an effect (OR = 0.94, *P* = 0.89).

This suggests that non-adherence to the above guidelines through incomplete outcome specification could allow investigators a large degree of flexibility in choosing which outcome definition to present. Greater adherence to the SPIRIT and ClinicalTrials.gov guidelines would reduce this risk. However, it is also important to highlight that explicit outcome definition is not always entirely straightforward and what is clear to one person may not be clear to another. For example, in TRIGGER, defining what constitutes a further bleeding event so there is no ambiguity whatsoever could be challenging. To prevent any ambiguity regarding the outcome definition, one approach could be to provide the computer code that will be applied to the database extracts to derive the final outcome. For example, in the TRIGGER protocol we could have specified the Stata code we planned on using to derive the further bleeding outcome, based on the data extracted from the trial database.

We note there were several limitations to this study. First, this is a re-analysis of only one trial in a specific therapeutic area and results may therefore not be generalisable to other trials. Second, the primary aim of the TRIGGER trial was related to feasibility, as opposed to clinical effectiveness; nonetheless, the primary clinical outcome was further bleeding and the example remains relevant to all outcome measures.

## Conclusions

We have demonstrated that modifications of an outcome definition led to substantially different treatment effect estimates in the re-analysis of a previously published clinical trial. These results highlight the importance of pre-specifying clinical trial outcomes in sufficient detail a priori, as failure to do so could lead to outcome switching and different definitions being analysed, with presentation of only the most favourable results.

## Abbreviations

RBC: Red blood cell
RCT: Randomised control trial
TRIGGER: Transfusion in gastrointestinal bleeding
